# The Editor's Letter-Box

**Published:** 1913-08-09

**Authors:** Sydney Holland

**Affiliations:** Chairman


					[Copy.]
London Hospital, Whitechapel, E.,
^ July 31, 1913.
he Society for the State Registration of Trained
Nurses.
irie EAR ?^adam'?I am uinch obliged to you for sending to
' after you had sent it to all the other members of the
0lnmittee of the London Hospital, a copy of the resolution
^ d by the Society of which you are the Hon. Secretary.
s it a joke, or is it meant seriously?
? * the latter, does your Society really think that the
j ^^ittee of the London Hospital is the least likely to he
. fenced by a resolution passed at a meeting with no one
1 t?1 Hospital present ?
he Committee of the London Hospital and, what is
?re important, the public and the medical profession, are
1 ectly satisfied with the training of the London Hos-
tli nurses, whose reputation, training, and skill only
r Society would venture to question. And considering
0 are the active leaders of that Society, this is no matter
surprise.
a London Hospital Committee are not likely to attach
atte ni?re importance to a resolution of your Society
St to dictate to them, than did the management of
artholomew's.?Yours faithfully,
At,* .
(Signed)
Sydney Holland, Chairman.
lss Margaret Breay.

				

